# Comparative sequence analysis reveals regulation of genes in developing schistosomula of *Schistosoma mansoni* exposed to host portal serum

**DOI:** 10.1371/journal.pone.0178829

**Published:** 2017-06-16

**Authors:** Wander de Jesus Jeremias, Flávio Marcos Gomes Araújo, Fábio Ribeiro Queiroz, Fabiano Sviatopolk Mirsky Pais, Ana Carolina Alves de Mattos, Anna Christina de Matos Salim, Paulo Marcos Zech Coelho, Guilherme Correa Oliveira, John Robert Kusel, Renata Guerra-Sá, Roney Santos Coimbra, Élio Hideo Babá

**Affiliations:** 1 René Rachou, Oswaldo Cruz Foundation – FIOCRUZ-MG, Belo Horizonte, Minas Gerais, Brazil; 2 Centro Universitário de Belo Horizonte – UNIBH, Belo Horizonte, Minas Gerais, Brazil; 3 Instituto Tecnológico Vale, Belém, Pará, Brazil; 4 Glasgow University, Centre for Open Studies, Glasgow, United Kingdom; 5 Federal University of Ouro Preto, Institute of Exact and Biological Sciences, Ouro Preto, Minas Gerais, Brazil; George Washington University School of Medicine and Health Sciences, UNITED STATES

## Abstract

Once inside a vertebrate host after infection, individual schistosomula of the parasite *Schistosoma mansoni* find a new and complex environment, which requires quick adjustments for survival, such as those that allow it to avoid the innate immune response of the host. Thus, it is very important for the parasite to remain within the skin after entering the host for a period of about 3 days, at which time it can then reach the venous system, migrate to the lungs and, by the end of eighth day post-infection, it reach the portal venous system, while undergoing minimal changes in morphology. However, after just a few days in the portal blood system, the parasite experiences an extraordinary increase in biomass and significant morphological alterations. Therefore, determining the constituents of the portal venous system that may trigger these changes that causes the parasite to consolidate its development inside the vertebrate host, thus causing the disease schistosomiasis, is essential. The present work simulated the conditions found in the portal venous system of the vertebrate host by exposing schistosomula of *S*. *mansoni* to *in vitro* culture in the presence of portal serum of the hamster, *Mesocricetus auratus*. Two different incubation periods were evaluated, one of 3 hours and one of 12 hours. These time periods were used to mimic the early contact of the parasite with portal serum during the course of natural infection. As a control, parasites were incubated in presence of hamster peripheral serum, in order to compare gene expression signatures between the two conditions. The mRNA obtained from parasites cultured under both conditions were submitted to a whole transcriptome library preparation and sequenced with a next generation platform. On average, nearly 15 million reads were produced per sample and, for the purpose of gene expression quantification, only reads mapped to one location of the transcriptome were considered. After statistical analysis, we found 103 genes differentially expressed by schistosomula cultured for 3 hours and 12 hours in the presence of hamster portal serum. After the subtraction of a second list of genes, also differentially expressed between schistosomula cultured for 3 hours and 12 hours in presence of peripheral serum, a set of 58 genes was finally established. This pattern was further validated for a subset of 17 genes, by measuring gene expression through quantitative real time polymerase chain reaction (qPCR). Processes that were activated by the portal serum stimulus include response to stress, membrane transport, protein synthesis and folding/degradation, signaling, cytoskeleton arrangement, cell adhesion and nucleotide synthesis. Additionally, a smaller number of genes down-regulated under the same condition act on cholinergic signaling, inorganic cation and organic anion membrane transport, cell adhesion and cytoskeleton arrangement. Considering the role of these genes in triggering processes that allow the parasite to quickly adapt, escape the immune response of the host and start maturation into an adult worm after contact with the portal serum, this work may point to unexplored molecular targets for drug discovery and vaccine development against schistosomiasis.

## Introduction

Schistosomiasis is a chronic, parasitic disease caused by trematode worms of the genus *Schistosoma*. At least 230 million people are infected by species of Schistosoma, and require treatment for schistosomiasis, worldwide every year. Schistosomiasis transmission has been reported in 77 countries, 52 of which are at the highest risk for infection. The disease is prevalent in tropical and sub-tropical areas, and especially in poor communities without access to clean water and adequate sanitation [1]. It is estimated that at least 90% of infected people requiring treatment for schistosomiasis live in Africa. In Brazil, *S*. *mansoni* is the prevalent species causing the chronic intestinal form of the disease and, at least, 25 million individuals live at risk of infection [2,3]. During its life cycle, *S*. *mansoni* establishes complex relationships with snails from the genus *Biomphalaria*, intermediate host, and its definitive mammalian hosts, which includes humans.

That *S*. *mansoni* schistosomula only begin to increase in biomass when they reach the vascular portal system of the definitive host was reinforced by evidence that individuals trapped in the pulmonary blood vessels remain immature for months after infection. In contrast, those that reach the portal system grow exponentially [4]. After this finding, it was shown that, when directly inoculated into the portal vein of the host, *in vitro* transformed schistosomula started egg laying around seven days prior to that of parasites derived from natural transcutaneous infection [5,6]. This period of 7 days corresponds to the time needed for schistosomula to migrate from skin to the liver-portal system, while passing through the lungs, during natural transcutaneous infection [7]. A subsequent study elicited a significant increase in cell proliferation of schistosomula derived from mice, after they were cultured in the presence of human portal serum, compared to the parasite of the same larval stage cultured in presence of human peripheral serum [8,9]. These findings suggest potential relationship with the presence of some constituent of the portal serum, which is absent in the peripheral serum. However, no specific molecule(s) was (were) indicated as responsible for the findings of the aforementioned studies.

The rate of growth and the cellular differentiation achieved by the parasite when it reaches the host liver portal blood system represents an attractive target for study, since controlling these processes may provide effective ways to prevent the progression of the disease to a severe form. Differentially expressed genes important for processes like host percutaneous penetration by cercariae, migration of intravascular forms of the parasite and, mating of adult worms within the host portal system, have already been explored for the development of approaches with new drugs, with the aim of treating schistosomiasis effectively [10].

A recent study [11] showed that among all the genes expressed by *S*. *mansoni*, only a small set of 38 are differentially expressed when the schistosomula mechanically transformed [12], compared to those that are skin transformed[13], which means that they are transcriptionally equivalent. This finding validates the results of previous gene expression studies where mechanically transformed schistosomula were used as representative of those transformed in the skin.

Current knowledge regarding cellular and molecular mechanisms that govern host-parasite interactions may be applied to the discovery of new drugs or vaccines that are effective against early stages of this parasite. In the present work, we used an Applied Biosystems SOLiD system NGS to study transcriptomes of schistosomula from *S*. *mansoni*, in order to identify differentially expressed genes which can be related to parasite development in the host portal system.

## Materials and methods

### Biological material

All schistosomula used in the present study were obtained through *in vitro* mechanical transformation from *S*. *mansoni* cercariae of the LE strain. Both, peripheral and portal sera were obtained from hamsters—*Mesocricetus auratus*, golden strain, grown in vivarium of the Rene Rachou Research Center—Oswaldo Cruz Foundation (CPqRR/FIOCRUZ). Oswaldo Cruz Foundation Ethic in Animal Use Committee (CEUA/FIOCRUZ) granted the license for animal use, under the register number: LW42/10.

### Hamster serum

Hamster peripheral serum was obtained through heart puncture next to left coronary artery. On average, 4 ml of peripheral blood was withdrawn from each animal, which yielded about 1.5 ml of serum. Portal serum was obtained after dissection and puncture of the portal vein of different animals than those used for peripheral blood sampling. The maximum volume withdrawn from this puncture was 1 ml of blood; this smaller volume was taken to assure that only portal blood was collected, without dilution with peripheral blood. Prior to both withdrawal procedures hamsters were anaesthetized with an intraperitoneal injection of 40mg/kg sodium thiopental (Thiopentax—Cristalia). The whole blood was left for 1 hour at room temperature to coagulate prior to serum being obtained by centrifugation at 850 x g for 5 minutes, followed by aspiration of the red blood cell free supernatant. The serum was then heated at 56°C for 30 minutes to inactivate the blood complement system. Portal blood from 35 hamsters was combined to compose a serum sample, which was used to supplement media of all schistosomula cultures. Likewise, for the peripheral serum, blood from 15 hamsters was combined. Samples with a final volume of approximately 20 ml for each serum were obtained from every repetition of blood withdrawal.

### Cercariae transformation and culture of schistosomula

Schistosomula were obtained through mechanical transformation of cercariae, as previously described [14], using the LE strain of *S*. *mansoni*. Schistosomula were cultured at 37°C in an atmosphere of 5% CO_2_, on MEM-HEPES medium (12,5g/L Glasgow Minimum Essential Medium—Sigma Aldrich, catG6148; 20mM HEPES—Sigma Aldrich, catH3375; 0.1% hydrolyzed Lactalbumin—Sigma Aldrich, cat61300; 0.1% anhydrous glucose Merck Millipore, cat346351; 0.5μM hypoxanthine—Sigma Aldrich, catH0888; 1μM hydrocortisone—Sigma Aldrich; 0.5% of 100x MEM Vitamin solution—Sigma Aldrich, catM6895; 1% Penicillin-Streptomycin mix—Sigma Aldrich, catP4333), supplemented with 10% (v/v) of hamster peripheral or portal serum.

Parasites were cultured in four different conditions: SPE3H –schistosomula incubated in peripheral serum for 3 hours; SPE12H –schistosomula incubated in peripheral serum for 12 hours; SPO3H –schistosomula incubated in portal serum for 3 hours; and SPO12H –schistosomula incubated in portal serum for 12 hours. Limited by the amount of peripheral and portal serum available, every individual repetition of the culture experiment used, four plates of 6 wells, with 6 ml of MEM-HEPES medium supplemented with 10% sera (v/v) per well. Each well had 4.0E+03 mechanically transformed schistosomula, so that each sample for RNA extraction, library construction and sequencing had 9.6E+04 schistosomula of a specific culture condition.

### RNA extraction and rRNA depletion

After each respective culture time (SPO3H, SPE3H, SPO12H and, SPE12H), parasites were collected and washed twice with MEM-HEPES medium and immediately submitted to RNA extraction. Total RNA extraction and isolation was performed with TRIzol Reagent (Invitrogen, cat15596-026) and, RNeasy (Qiagen, cat74106), with a final treatment with DNAse Turbo (Ambion, catAM1907), all according to the procedures recommended by manufactures. The rRNA was depleted using RiboMinus Eukaryote Kit for RNAseq (Invitrogen, cat10837-08). Before rRNA depletion, mRNA quality was verified by measuring rRNA integrity through capillary electrophoresis in a Bioanalyzer 2100 (Agilent, catG2939AA), employing RNA 6000 Pico Reagents (Agilent, cat5067-1514). Quantitation of mRNA was assessed by using a Qubit 2.0 Fluorometer (Life Technologies catQ32866) and a Qubit RNA Assay Kit (Invitrogen catQ32852) for RNA samples, according to the protocol recommended by the manufacturer.

### Preparation of cDNA libraries and sequencing

RNA sequencing was performed using a SOLiD Total RNAseq system Kit (Applied Biosystems, cat4445374), following the procedures recommended by the manufacturer. After reverse transcription using random primers, the synthesized cDNA molecules were size-selected for fragments between 150 and 250 bp, and quantified with a Qubit 2.0 Fluorometer (Life Technologies catQ32866). The cDNA was diluted to a final concentration suitable for monoclonal amplification by emulsion-PCR, while copies of template molecules were attached to beads. Beads were deposited onto slides upon which the sequencing reactions, as well as washing steps, were performed automatically in the 5500 SOLiD platform. The 5500 SOLiD sequencer was used to read short DNA sequences (50nt long), from the DNA attached to the beads with 50 rounds of oligonucleotide annealing and ligation (five different primers, 10 sequential ligations per primer).

### Mapping

Reads in color-space format were mapped against the fifth version of the *S*. *mansoni* reference genome [15], using Bowtie aligner [16] with adjusted parameters [11]. Reads that mapped to ribosomal and globulin sequences were excluded from further analysis; only uniquely mapped reads were considered and used for downstream analysis.

### Differential expression

Differential expression was assessed by using transcript abundance count tables as input to the DESeq2 package [17] in the R environment [18]. Differentially expressed transcripts were analyzed among replicates of the libraries generated from the 3 hour cultures against those of the 12 hour cultures of the same serum (peripheral or portal). Transcripts with adjusted *P values* lower than 0.05 were considered differentially expressed. In the final list of differentially expressed transcripts, one was considered up-regulated only if they exhibited a Log2Change fold higher than 1, and down-regulated if Log2Change fold was lower than -1 [19].

### Gene ontology (GO) enrichment analyses

GO enrichment analysis was performed with Blast2GO, software available at https://www.blast2go.com/blast2go-pro/download-b2g.

### Validation of differentially expressed genes by real time polymerase chain reaction (RT-qPCR)

The relative expression of a subset of the genes found deferentially expressed between SPO3H and SPO12H was determined with RT-qPCR. Primer 3 software [20] was used to design the primers and probes for each gene. The oligos were acquired from Thermo Fisher Scientific (Custom TaqMan Gene Expression Assay), Brazil (sequences available in S1 Table). First strand cDNA was synthesized from 1μg of total RNA samples by using a High Capacity cDNA Reverse Transcription Kit (Thermo Fisher Scientific, Brazil—cat4368814) according to the manufacturers recommendations. The qPCR reactions were performed using a ViiA 7 Real-Time PCR System, with 384-well block equipment and TaqMan Universal PCR Master Mix (Thermo Fisher Scientific). Primer efficiencies were calculated using 10-fold dilutions of a freshly transformed schistosomula cDNA. The gene for cytochrome oxidase subunit 1 (COX1) was used as a control for relative expression of a given gene through the calculation of the delta-cycle threshold (Ct). Freshly transformed schistosomula, without additional culture or contact with portal or peripheral serum, were used as a control for culture condition. The delta-delta-Ct method was used to calculate the fold change of expression of a target gene between the delta-Ct of the experimental conditions (SPO3H and SPO12H) and the control condition (schistosomula freshly transformed). The fold change values obtained are means of three biological replicates.

## Results

### Parasite culture and RNA isolation for library preparation

The mechanically transformed schistosomula were inspected for the presence of tails or cercariae remaining from the transformation procedure; only preparations with less than 5% of this contamination were cultured in presence of hamster sera. Under light microscopy, the schistosomula cultured in presence of peripheral and portal serum appeared similar. Approximately 80,000 schistosomula were obtained from each preparation, which yielded around 5–8μg of total RNA. The amount of mRNA needed to construct each library was obtained from material of at least two preparations, with the rRNA integrity having been checked by capillary electrophoresis (BioAnalyzer 2100, Agilent Technologies).

### Sequencing

Approximately 15 million of 50 nt short reads generated for each library were mapped against the fifth version of the *S*. *mansoni* genome. Table 1 summarizes the number of reads filtered at different levels. Unmapped reads, as well as those mapped against ribosomal or transporter RNA sequences in the reference genome were excluded. Only reads mapped once at the genome, and with high scores of alignment, were considered for analysis. Since for each sample, approximately 15 million of 50 nt reads were correctly mapped, they covered about 45.5x the predicted transcriptome of *S*. *mansoni* [15].

**Table 1:** Summary of filtering of RNASeq reads.

Sample	Replicate	SOLID ID	Processedreads	Mapped reads
SPO3H	1	SPO_3H0	22,593,851	12,862,079 (56.93%)
	2	SPO_3H1	44,195,718	16,449,088 (37.22%)
	3	SPO_3H2	39,039,144	14,601,483 (37.40%)
SPO12H	1	SPO12_12H0	21,124,149	12,570,187 (59.51%)
	2	SPO12_2H1	40,505,621	14,860,486 (36.69%)
	3	SPO12_12H2	36,698,989	13,448,911 (36.65%)
SPE3H	1	SPE_3H0	23,249,600	13,588,324 (58.45%)
	2	SPE_3H1	45,458,544	17,539,615 (38.58%)
	3	SPE_3H2	43,185,497	16,955,168 (39.26%)
SPE12H	1	SPE12_12H0	23,509,190	13,667,938 (58.14%)
	2	SPE12_2H1	40,341,711	16,303,097 (40.41%
	3	SPE12_12H2	44,680,174	17,471,587 (39.10%)
			Mean	15,026,496.9(44.86%)

SPO3H—schistosomula cultured for 3 hours in portal serum; SPO12H—schistosomula cultured for 12 hours in portal serum; SPE3H—schistosomula cultured for 3 hours in peripheral serum; SPE12H—schistosomula cultured for 12 hours in peripheral serum;

The raw sequence data was uploaded in the Sequence Read Archive (SRA) of the National Center for Biotechnology Information (NCBI), and is fully available under the Bioproject number PRJNA362725 and the ID number 362725.

### Genes differentially expressed between SPO3h and SPO12h

**Fig 1:**
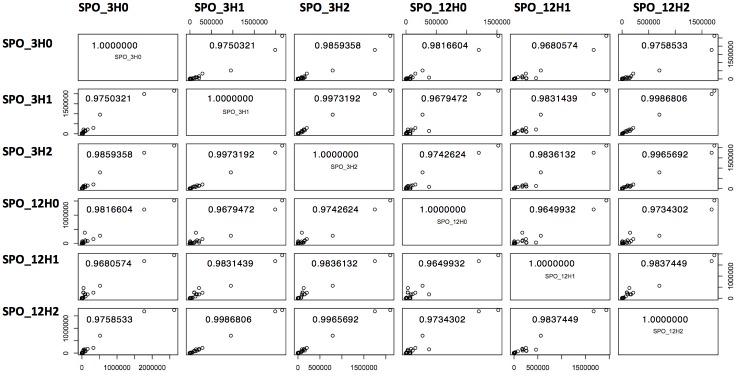
Similarity between schistosomula cultured for 3hours (SPO3h) and for 12hours (SPO12h) in the presence of hamster portal serum. Pearson’s correlation coefficients for gene expression values were high for all paired samples.

After mapping the short reads with Bowtie, alignments were computed in order to generate a count table with 11.844 rows representing all the transcripts annotated in the 5.0 version of the genome of *S*. *mansoni* [15]. We found 11,103 transcripts with detectable expression in at least one of the samples. Correlation analysis produced high values for Pearson’s coefficient and Spearman’s rank correlation for all possible paired combinations, which confirmed the high degree of similarity among the samples (Fig 1). Through the ggplot package in R environment, multiple scatter plots were constructed facilitating pairwise comparisons of gene expression levels between the libraries (Fig 1) with their respective Pearson’s coefficient.

**Fig 2:**
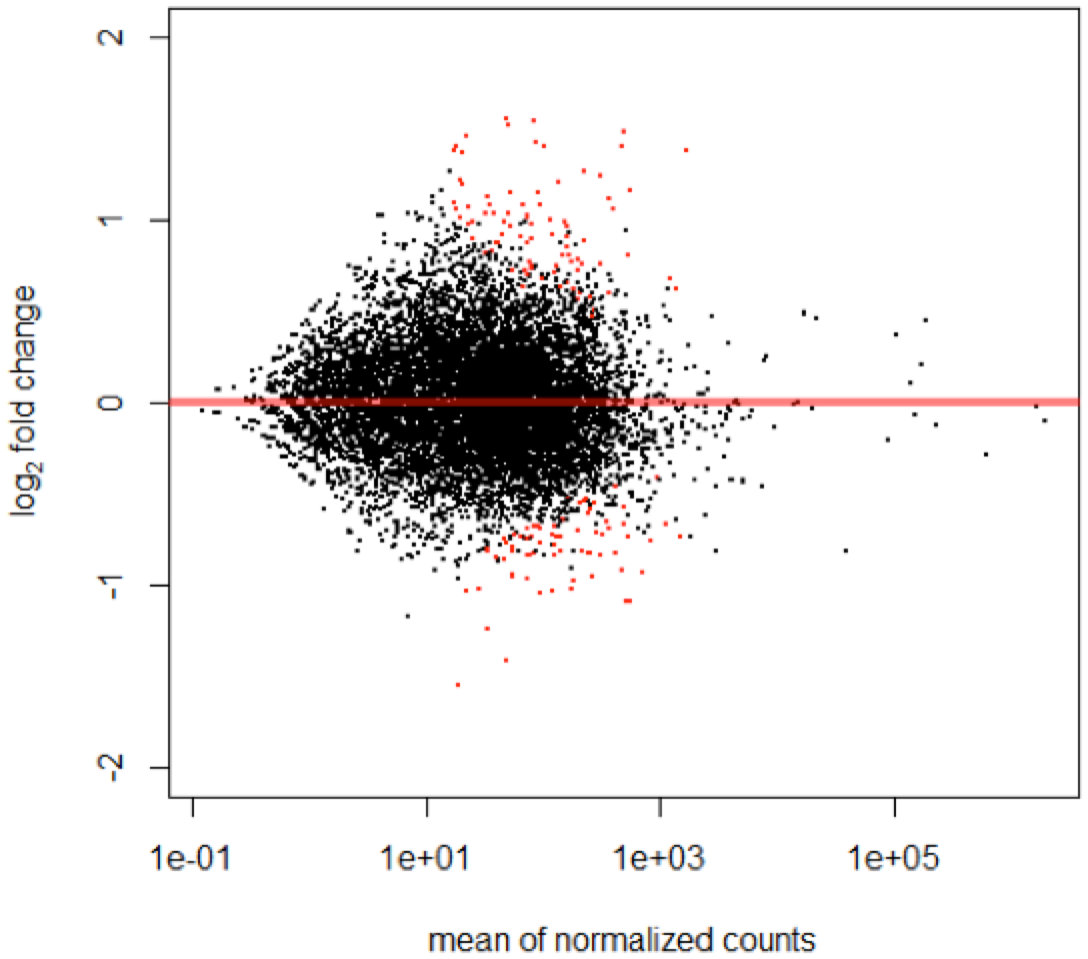
Differential gene expression between SPO3h and SPO12h. The MA plot of mean expression, that is the average of counts normalized by size factor (x-axis) plotted against Log2change fold 9y-axis), between schistosomula SPO3h and SPO12h (adjusted p-value < 0.05). Transcripts with positive Log2Change fold are up-regulated in schistosomula after 12hours of exposure to hamster portal serum, and those with negative Log2Change fold are up-regulated after 3hours exposure to the portal serum. The red spots represent transcripts with p-values < 0.1, as default.

Once the high correlation among samples of all the conditions was confirmed, differential expression analysis was performed with the DESeq2 package [17], also in the R environment. One hundred three transcripts differentially expressed between SPO3h and SPO12h were identified (adjusted *p value* < 0.05). Sixty five transcripts were up-regulated (change fold > 1), and thus highly expressed after 12 hours of culture, while 38 were down-regulated (change fold < 1), and therefore minimally expressed after 12 hours. A graphical representation of the general gene expression (MA plot) is shown in Fig 2 (change fold in logarithmic scale *versus* global average of the normalized count of reads). The complete list of change fold values of the transcripts and their respective adjusted *p-values* are provided in S2 Table.

The list of 103 differentially expressed genes was further filtered by a second list of genes also differentially expressed in a similar comparison between schistosomula cultured in the presence of hamster peripheral serum for the same period of time, 3hours (SPE3H) and 12hours (SPE12H). Genes present in both lists were excluded from the analysis. Thus, 58 genes were finally considered exclusively expressed in portal, but not in peripheral serum.

A subset composed of 17 genes (change fold > 1 or < 1) were also evaluated with qPCR (Table 2). According to current annotation, these genes are involved in membrane transport, response to stress, signaling, cytoskeleton development, and protein and nucleotide synthesis, among others. The fold change values from both methods exhibited, at the 95% confidence level, the same tendency of up- or down-regulation for15 of the 17 genes (Smp024390.1, Smp157070.1, Smp134570.1, Smp130260.1, Smp083080.2, Smp089000.1, Smp103560.1, Smp079230.1, Smp019060.1, smp042790.1, Smp079420.1, Smp007070.1, Smp136310.1, Smp145540.1 and, Smp175790.1). Only two genes (Smp027990.1 and Smp177040.1) presented discordant tendency of expression between the methods of RNAseq and qPCR.

**Table 2:** Subset of genes differentially expressed in schistosomula cultured of 12hours in portal serum by RNASeq.

Gene identifier	Description	Adj. P-value	Fold Change
Smp_157070	cysteine rich with egf domains protein	1.67E-03	2.76
Smp_134570	hypothetical protein	2.10E-05	2.7
Smp_024390	microsomal signal peptidase 25 kDa subunit	4.02E-03	2.64
Smp_130260	hypothetical protein	6.41E-03	2.6
Smp_177040	gpi mannosyltransferase 2	9.39E-04	2.16
Smp_083080	Activator of 90 kDa heat shock protein ATPase	1.10E-04	2.12
Smp_027990	homeobox protein nk 2	5.74E-03	2.11
Smp_089000	translocon associated protein subunit delta	2.73E-02	2.05
Smp_103560	hypoxanthine guanine phosphoribosyltransferase	4.27E-02	2.05
Smp_079230	immunophilin FK506 binding protein FKBP12	4.68E-02	2.03
Smp_079420	ankyrin repeat domain containing protein 42	5E-02	2.02
Smp_042790	dolichol phosphate mannosyltransferase	5E-02	2.02
Smp_019060	sec61 beta subunit	1.67E-03	2.01
Smp_136310	sodium bile acid cotransporter	2.85E-02	0.49
Smp_007270	Smoothelin	4.34E-03	0.49
Smp_175790	phospholipid translocating ATPase	7.63E-04	0.42
Smp_145540	muscarinic acetylcholine receptor	7.16E-04	0.34

Differentially expressed genes with adjusted p-value <0.05.

**Fig 3:**
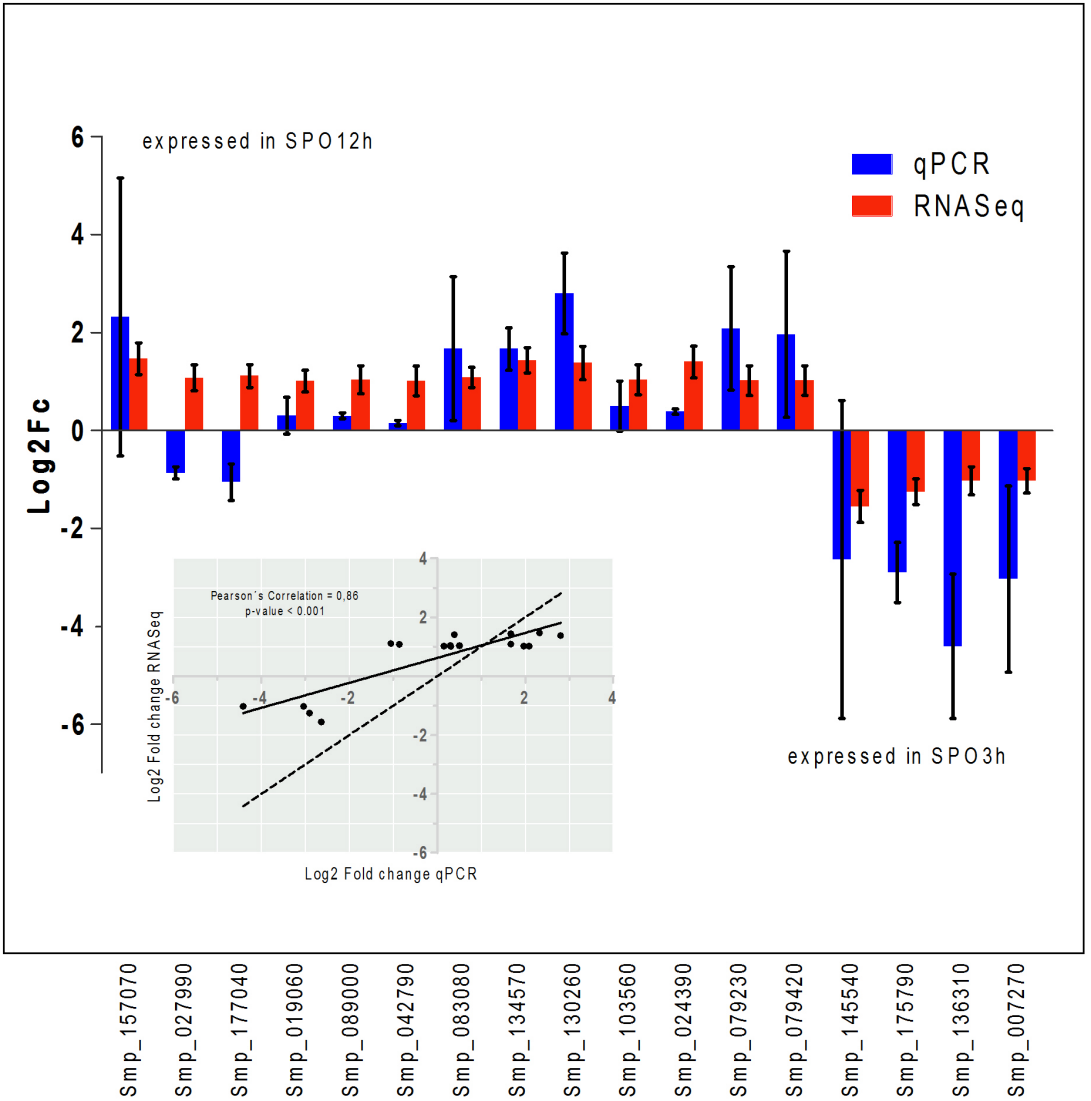
Relative expression of a subset of genes as determined by RNASeq and qPCR assays. Bar plots of Log2ChangeFold of the subset of genes chosen to validate the RNASeq (red) pattern of expression by using qPCR (blue). The Pearson’s coefficient, at a 95% confidence level is shown on the graph and illustrates the gene expression assessed by both methods. The scatter plot shows the strong correlation of gene expression calculated by the two methods (Pearson’s correlation = 0.86, p-value < 0.001). The solid line is the linear regression adjusted to the points of correlation; the dashed line represents a correlation where x = y.

Fig 3 compares the fold changes generated by the RNAseq and qPCR analyses for the 17 genes. Pearson’s coefficient (*p-value* <0.05), between each assay, indicates a strong correlation (0.86), as 15 of the 17 targets were in agreement with the direction of the change fold.

## Discussion

Schistosomula have been used in transcriptome studies since the era of DNA microarrays [10, 21, 22], and until more recent times when NGS techniques [11, 23, 24] are used. These studies compared gene expression among schistosomula submitted to different lengths of time of culture and at different lifecycle stages of the parasite, but none of them attempted to simulate the intravascular condition of a vertebrate host. In one study in this sense [25], the pattern of gene expression of schistosomula cultured for 3 hours in presence of erythrocytes of a vertebrate host was compared to those kept for 5 days in presence of these cells. By using a DNA microarray containing 12,166 sequences of different *S*. *mansoni* contigs, the authors inferred the main changes in gene expression were result of ontologies related to blood feeding, cell adhesion, cytoskeletal development and stress response.

In the present work we investigated transcriptional changes that occur during a specific stage of the *S*. *mansoni* life cycle, the schistosomula, when it reaches the portal venous system of a mammalian host. Two particular time periods were selected for this study, which represent the early contact of the parasite with an important constituent of the portal venous system, the serum and its soluble molecules. In this location, it is well known that the parasite has experiences a tremendous increase in biomass followed by development into the mature stage [4,6]. Once in the adult stage, parasites mate and the female starts laying eggs, which is essential in determining several aspects of the pathogenesis of schistosomiasis. This work aimed to identify the genes potentially associated with the morphological changes that occur during development of this last stage of this parasites life cycle.

We were able to identify differentially expressed genes between 3 hours and 12 hours of incubation within hamster portal serum. A high degree of similarity in gene expression among libraries of the same condition was observed, which strengthens the findings reported in this manuscript. Features of the Gene Ontology (GO) hierarchy of differentially expressed genes highlighted terms such as membrane transport, response to stress, signaling, cytoskeleton development, protein and nucleotide synthesis, among others which, coupled with the literature concerning parasite growth, are discussed as follows.

### Surface membrane

In newly transformed schistosomula, the ability to modify the surface membrane through acquisition of host molecules represents the formation of a host-parasite interface, at which skin-derived factors may stimulate internal membrane receptors to drive important actions of parasite development and facilitate escape from the host immune system [26, 27, 28, 29, 30]. In the present study, a considerable number of the genes up-regulated after 12 hours of stimulus by portal serum were related to membrane activity, mainly regarding the transport of substances, both on the cell membrane as well as in internal organelles.

A previous study [31] showed that in newly transformed schistosomula, the parasite surface, gut, and sub-tegumental structures all have an increase in phospholipid uptake activity. Furthermore, short chain phosphatidylcholine analogs (C_6_-NBD-PC and C_5_-Bodipy-PC) are taken up internally while a longer chain analog (C_12_-Bodipy-PC) was viewed only on the surface of the parasite, in the gut, and occasionally in the acetabulum, but not in the sub-tegumental location. The Smp_175790.1—*phospholipid-translocating ATPase*, an up-regulated gene, may be involved in the transport of phospholipids across the membrane. Such activity may be related to the well-known role of phospholipid transport in generating or maintaining lipid asymmetry across the phospholipid membrane bilayer [32, 33]. Proteins sharing the same type of activity (ABC transporters) were identified as being potentially involved in schistosome susceptibility or resistance to drug treatment [34]. Moreover, these proteins may be involved in host phospholipid acquisition by the parasite into sub-tegumental compartments, which is important in regulating phospholipid biosynthesis and transport, particularly of lipid components on the surface membranes, thereby allowing the parasite to adapt and escape from the host immune system.

The N-glycan synthesis is another transcriptionally active process, as showed by the up regulation of the gene Smp_042790.1—*dolichol phosphate mannosyltransferase*. This enzyme is usually involved in the biosynthesis of the N-glycan core in endoplasmic reticulum (ER) of eukaryotes [35]. Evidence that parasite-derived glycans are both bioactive and immunogenic suggests that they play important roles in immunomodulation, as well as in evasion from the host immune response [36]. The increase in the activities of phospholipid translocation and N-glycan synthesis after stimulus by portal serum may be a determinant of increased membrane permeability, similar to that observed in the *in vivo* transformed schistosomula of our previous work [37]. This may increase the ability of the parasite to evade the immune responses of the host by changing the repertoire of molecules exposed on the external membrane.

### Endoplasmic reticulum (ER)

According to our data, the ER-associated protein degradation pathway (ERAD) is enhanced in response to early contact with hamster portal serum. We identified up-regulated genes coding for two subunits of the translocon-associated protein (TRAP) complex: Smp_019060.1—*sec61 beta subunit* and Smp_089000.1—*translocon associated protein subunit delta gene*. Data generated from studies with mammalian cells [38, 39] suggest that gene ortologs of the sec61 complex recruits the TRAP complex. The products of the two genes act together to form a membrane channel associated with the ribosome during co-translational protein translocation. Gene ortologs of Smp_059350.1—*der1 protein*, a Derlin’s family protein from mammal, is also implicated in the retro-translocation of protein from ER to cytosol [40, 41,42]. Proteins that fail ER quality control after translation are retro-transported to cytosol and finally degraded by ubiquitin-proteasome machinery [43, 44]. Both, yeast and mammalian gene ortologs of Smp_157360.1 E3—*ubiquitin protein ligase synoviolin B* are among the ubiquitin ligases required for ERAD and are members of large protein complexes, which include components active in substrate-recognition, targeting and retrotranslocation. Since this transcript is also up-regulated in schistosomula after portal stimulus, important role of its product in the protein degradation may be suggested, as a previous study has been proposed that this enzyme is involved in formation of retrotranslocation channels [45], housing two activities: ubiquitylation and retrotranslocation.

The fact that ERAD is active mainly to degrade newly synthesized proteins that failed ER quality control is aligned with increased activity of translation after portal serum stimulus, as is reinforced by the genes up-regulation of some genes that function in ribosome assembly and also in signal protein recognition. Three genes, Smp_119920.1—*ribosomal protein S16*, Smp_032760.1—*ribosomal protein S11* and Smp_031570.1—*ribosomal protein L18*, are annotated as “structural constituents of ribosome” at the level of molecular function GO, showing that the organelle has the conformational structure to let protein synthesis. Further, the correct addressing of newly synthesized proteins to ER is active once the genes Smp_207010.1—*signal recognition particle 72 kDa subunit* and Smp_024390.2—*microsomal signal peptidase 25 kDa subunit* are up-regulated. The signal recognition particle (SRP) has a pivotal role in targeting secretory proteins to the membrane of the rough endoplasmic reticulum (RER) when assembled with other subunits of the SRP complex, and also interacts with the docking protein of the ER membrane [46]. It also may participate in slowing protein synthesis, which facilitates the coupling of protein translation to the translocation process. Signal peptidase is part of a protein complex essential for cleavage of the signal peptides of most of the secretory or membrane proteins as soon as its translocation from the cytoplasmic side to the ER lumen is completed, which ensures the newly-synthesized proteins are correctly addressed [47].

Another gene found up-regulated, which is also related to activity within the ER, is Smp_155510.1—*transmembrane protein66*. Although the function of the protein 66 (*TMEM66*) is not well known, it was recently functionally identified as a calcium homeostasis regulator, due to its role in down-regulation of store-operated calcium entry (SOCE), a process of calcium influx that occurs in response to depletion of ion stores in ER [48].

Two up-regulated genes with activity on the Golgi apparatus, Smp_167230.1—*golgi resident protein gcp60* and Smp_072950.1—*transmembrane emp24 domain containing protein 4*, suggest that the maintenance of organelle structure is affected by the 12hour stimulus with host portal serum, as is the protein traffic between the ER and the Golgi [49]. Additional evidence [50, 51] also suggests that they may be involved in controlling apoptosis in yeast and human.

Taken together, the findings discussed above highlight that after a short stimulus (12 hours) by host portal serum, the activity in ER is enhanced toward protein synthesis. This is likely, in response to external signals, which increase the protein repertoire important for parasite gain of biomass, and its development to the adult worm stage. In addition to protein synthesis, protein degradation also occurs, showing that a turnover of these molecules is also occurring and may be acting as part of the parasites mechanism of scape from the host immune response.

### Response to stress

Components of the portal serum probably cause stress to the parasites, therefore demanding an adaptive response. This is especially true, considering that when a parasite accesses the portal venous system of the host during the course of infection, it finds a new environment where active immune cells represent a challenge to be overcome. Exposure of the parasite exposure to the broad range of molecules present in the blood of the host body portion, like nutrients and metabolites, is still worthy of consideration. The increased expression of the superoxide dismutase Smp_176200.2—*superoxide dismutase Cu-Zn* suggests that the parasite activates its mechanisms in order to prevent damage from oxidative stress. Previous research [52] has already shown an increase in transcriptional activity of this gene in the adult worm stage in a gender-associated fashion, as well as strain -gender-associated differential expression [53], with the up-regulation of the gene in a Chinese and Philippine strain of female adult worms of *Schistosoma Japonicum*. Taken together, these findings highlight the possible involvement of the enzyme in detoxification of products from hemoglobin digestion in female adult worms as soon as egg laying starts. The gene was also previously shown to be up-regulated in response to oxidative stress in adult worms [54].

One constituent of the HSP complex, Smp_097380.1—*heat shock 10 kDa protein 1*, plus one *S*. *japonicum* homolog of the gene Smp_064860.1—*stress induced phosphoprotein 1*, which was predicted to directly interact with proteins from the heat shock complex (HSP) in adult worm tegument [55], were up-regulated. Considering the fact that in some instances, the ERAD process discussed above depends on active HSP proteins, we suggest that the unfolded proteins are retro-transported to cytosol and degraded by the proteasome in response to oxidative stress caused by 12 hours of contact with the constituents of the host portal serum.

A peptidase, Smp_040790.1—*peptidyl prolyl cis trans isomerase B*, whose activity was shown to be important for accelerating protein folding/refolding in the rate limiting slow step, by promoting the proline *cis*-*trans* isomerization [56, 57], was up-regulated. This peptidase is similar to cyclophilin proteins, which link to cyclosporine A to develop immunosuppressive action on mammal[58]. In other studies, cyclosporine A was shown to protect mouse and rat from schistosome infection [59, 60, 61, 62]. Cyclophylin has also been suggested to play an important role in mammalian metabolism, since its production is increased in murine splenocytes 72 hours after mitogenic stimulation [63]. Another peptidase was also shown to be up-regulated, the Smp_194090.1—*subfamily S1A unassigned peptidase—S01 family*, however, the product of this gene was recently identified as proteolytically inactive [64], since it lacks the serine residue in the catalytic triad—His, Asp and Ser typical of the S1 family [65], and so remains unclear.

### Signaling

Increase in transcriptional activity in response to host portal stimulus was expected, particularly for some of the genes that are determinants of the signaling that triggers proliferative activity, as previously reported after the same stimulus [7,8]. One gene for tyrosine kinase receptor (RTK), Smp_011700.1—*proto oncogene tyrosine protein kinase ROS*, was up-regulated, which is included in the family of the vascular endothelial growth factor—VEGF [66, 67], and appears to be an important stimulus of the proliferative activity trigged in the parasite when it is in contact with the portal serum. The importance of this gene is reinforced by the fact of that soluble egg antigen (SEA) causes up-regulation of VEGF, *in vitro*, and is an important factor for the increase in angiogenesis around the granuloma in the liver of a host infected by *S*. *mansoni* [68]. Further, the up-regulation of VEGF was also reported in the portal vein of rats in response to injuries caused by partial liver resection, along with other factors acting on liver regeneration [69]. Taken together, these findings suggest additional roles for VEGF as a stimulus for the proliferative activity in the parasite body as soon as it reaches the portal vein system of the host, instead of the previous reported action on host angiogenesis. Additional studies are going to be conducted by our group, aiming to determine the importance of the signaling triggered by this receptor during parasite development. Inhibitors of VEGF activity are going to be supplemented in culture media, also in presence of portal serum, and aspects of morphology will be evaluated at selected time periods for as long as possible during culture.

Two genes with signaling regulatory activity when transcription is increased, the Smp_027990.1—*homeobox protein nk2*, a neural development gene activator, and the Smp_157940.1—*nuclear inhibitor of protein phosphatase 1* were up-regulated. The NK2 domain is a highly conserved motif [70] that was identified in all vertebrate NK-2 class transcription factors and also in *Drosophila* NK2 transcription factors, and is expressed during the development of the central nervous system [71] and pancreas [72]. The nuclear inhibitor of protein phosphatase 1 –NIPP1 is included in the family of protein phosphatase 1 –PP1, regulatory proteins [73, 74] and was shown to be essential in spliceosome assembly [75]. An ortolog of the mammalian gene, when over-expressed in *Drosophila*, was cell-lethal to several tissues and developmental stages, probably due to its interaction with splicing factors through its highly conserved fork head-associated (FHA) domain [76]. Considering the possible importance of this gene to the development of the parasite nervous system, additional experiments will be planned to study its role in parasite maturation.

A protein containing a common protein–protein interaction motif, the Smp_079420.1—*ankyrin repeat domain containing protein 42*, was also found up-regulated after 12 hours of exposure to host portal serum. The ankyrin repeat domain is a 33 amino acid sequence that adopts a helix-loop-helix structure [77], which has been found in several proteins with a variety of different functions, comprising cell–cell signaling, transcription and cell cycle regulation, in addition to other cellular processes [78]. In schistosome, the ankyrin repeat domain was atypically found in the Casp C protein, a homolog of human caspase-8, and raised questions regarding its possible role in an alternative, still unknown, pathway to apoptosis [77].

The genes discussed here may be implicated in signaling regulation of transcription both in earlier activities, through growth factors, and in the final steps of RNA processing, in response to host portal serum stimulus.

Given the importance of signaling to a wide variety of activities in the parasite in response to its diverse environmental conditions during its life cycle, our group has particular interest in developing additional investigations using silencing techniques to explore the true roles in parasite development of the genes highlighted in this session as part of initial studies for the development of new drugs.

### Nucleus and nucleotide metabolism

Nuclear transport of phosphoprotein and RNA was shown to be active in response to portal serum stimulus. One member of the *Mex3* protein (the *muscle excess protein 3* [79]) group, the Smp_032280.1—*RNA binding protein MEX3B*, was up-regulated. In nematodes, the activity of Mex3 protein as a translational regulator, which helps maintain multiple morphogenetic potential in early embryonic germ lines, has been well established [80]. Human ortologs were characterized as related to RNA-binding phosphoproteins that shuttle between the nucleus and the cytoplasm and, according to their different final sites in the cytoplasm, may participate in mRNA storage, regulation of translation or degradation [81]. Furthermore, due to the ability to directly interact with the protein Argonaute, they are thought to be involved in RNA interference and miRNA biosynthesis pathways [82]. This last finding is corroborated by the up regulation of another gene with miRNA processing activity, an exportin-like protein, Smp_124820.1—*chromosome region maintenance protein*. Exportin acts on nuclear membrane protein transporting and the transport of different species of RNA to cytosol, and may participate in miRNA biogenesis in response to portal serum stimulus, thereby playing important roles in schistosomula development, as previously suggested [83].

The biosynthesis of nucleotides was active in response to portal serum stimulus, as the gene coding to for the main enzyme of the purine *salvage* biosynthesis pathway, Smp_103560.1—*hypoxanthine guanine phosphoribosyltransferase (HGPRT)*, was up-regulated. The importance of the enzyme hypoxanthine phosphoribosyl transferase and also guanine phosphoribosyl transferase in primarily supplying the parasite with guanine is well documented [84, 85], since the parasite does not express enzymes for a *de novo* pathway of purine synthesis [86]. This is one more activity reveled by our data that reinforces the role of the portal serum as a stimulus of the proliferative activity of the parasite body, once it reaches the venous portal system of the host.

### Miscellaneous

Some of the genes up-regulated after 12 hours of portal serum stimulus had no well-known function, and must be further studied, such as the *S*. *japonicum* ortolog of the gene Smp_011270.1 sj ts4 protein and an additional five other genes poorly annotated in genome of *S*. *mansoni* (Smp_202980.1 hypothetical protein; Smp_063330.1 hypothetical protein; Smp_024370.1 hypothetical protein; Smp_095400 hypothetical protein; Smp_130260 hypothetical protein; Smp_134570 hypothetical protein).

## Conclusions

The importance of portal serum in increasing cell proliferation in *S*. *mansoni* has been previously demonstrated [8,9], thus it is valuable to elucidate which genes are differentially expressed in the presence of this stimulus. There has been no previous study thoroughly clarifying the pattern of gene expression in the presence of portal serum. However, the presence of erythrocytes and the crossing of natural membrane barriers during infection, as well as the immune cells involved in the host response against parasite infection, were not por of our study, these are features need to be considered in the future.

Taken together, the genes discussed in our work corroborate the hypothesis that the parasite, when in contact with host portal serum for at least 12 hours, activates several different pathways that allow it to finally stimulate the proliferative (transcription and translation) and specification processes, such as stress response, and nervous system and muscle tissue development, important for its development into an adult worm. Our data corroborate the findings of a previous work where schistosomula were cultured in the presence of another intravascular component of the vertebrate host, namely erythrocytes [25]. These authors found up-regulation of some genes related to the processes of stress response, as well as cytoskeletal development, suggesting that these processes may work in synergy towards the extensive development of the parasite whenever it comes into contact with the entire composition of the fluid of the portal venous serum. These findings highlight some possible molecular targets for further research aiming to verify their true significance to the development of the parasite.

## Supporting information

S1 Table List of oligos used in qPCR of samples SPO3H and SPO12H. (DOCX)

S2 Table Differentially expressed genes of schistosomula cultured for 12hours in portal serum. (DOCX)

## References

[1] Steinmann P, Keiser J, Bos R, Tanner M, & Utzinger J. Schistosomiasis and water resources development: systematic review, meta-analysis, and estimates of people at risk. *Lancet Infect Dis*. 2006 6(7), 411–425. doi: 10.1016/S1473-3099(06)70521-7 16790382 10.1016/S1473-3099(06)70521-7

[2] Lambertucci JR, Acute schistosomiasis mansoni: revisited and reconsidered: Mem Inst Oswaldo Cruz, 2010, 105, 422–35. 20721485 10.1590/s0074-02762010000400012

[3] WHO Fact sheet No115, Jan 2012 –Schistosomiasis—http://www.who.int/mediacentre/factsheets/fs115/en/index.html#

[4] Barbosa MA, Pellegrino J, Coelho PMZ, Sampaio IBM. Quantitative aspects of the migration and evolutive asynchronism of *'Schistosoma mansoni'* in mice. Rev. Inst. Med. Trop. 1978 20: 225–233. 684324

[5] Coelho PMZ, Pellegrino J, Pereira LH, Mello RT. Migration of schistosomula (*Schistosoma mansoni*) collected from hamsters and inoculated intraperitoneally in mice. Trans R Soc Trop Med Hyg. 1976;70(2):161. 960200 10.1016/0035-9203(76)90181-4

[6] Rocha MO & Coelho PMZ. The importance of skin and pulmonary phase to the development of '*Schistosoma mansoni*' in albino mice Rev. Inst. Med. Trop. 1980 22:157–163. 7193903

[7] Faust EC, Jones CA, Hoffmam WA. Studies on schistosomiasis mansoni in Puerto Rico. III- Biological studies. 2. The mammalian phase of the life cycle. *Puerto Rico Journ Publ Healt Trop Med*. 1934; 10: 133–196.

[8] Shaker YM, Wu CH, el-Shobaki FA, Ashour E, Khattab HM, Draz HM et al Human portal serum stimulates cell proliferation in immature *Schistosoma mansoni*. Parasitology 1998, 117: 293–299. 9820850 10.1017/s0031182098003096

[9] Draz HM, Ashour E, Shaker YM, Khattab HM, Wu CH, Wu GY. Host susceptibility to schistosomes: effect of host sera on cell proliferation of *Schistosoma mansoni* schistosomula. J Parasitol. 2008 94(6):1249–52. doi: 10.1645/GE-1607.1 19127970 10.1645/GE-1607.1

[10] Fitzpatrick JM, Peak E, Perally S, Chalmers IW, Barrett J, Yoshino TP et al Anti-schistosomal intervention targets identified by lifecycle transcriptomic analyses. PLoS Negl Trop Dis. 2009 11 3;3(11):e543 doi: 10.1371/journal.pntd.0000543 . 19885392 10.1371/journal.pntd.0000543 PMC2764848

[11] Protasio AV, Dunne DW, Berriman M. Comparative study of transcriptome profiles of mechanical- and skin-transformed Schistosoma mansoni schistosomula. PLoS Negl Trop Dis. 2013;7(3):e2091 doi: 10.1371/journal.pntd.0002091 23516644 10.1371/journal.pntd.0002091 PMC3597483

[12] Brink LH, McLaren DJ, Smithers SR. Schistosoma mansoni: a comparative study of artificially transformed schistosomula and schistosomula recovered after cercarial penetration of isolated skin. Parasitology. 1977;74(1):73–86. 320543 10.1017/s0031182000047545

[13] Clegg JA, Smithers SR & Terry RJ. Acquisition of human antigens by *Schistosoma mansoni* during cultivation “in vitro”. Nature, 232:653–654, 1971 4999224 10.1038/232653a0

[14] Ramalho-Pinto FJ, Gazzinelli G, Howells RE, Mota-Santos TA, Figueiredo EA, Pellegrino J. Schistosoma mansoni: defined system for stepwise transformation of cercaria to schistosomule in vitro. Exp Parasitol. 1974 12;36(3):360–72. . 4139038 10.1016/0014-4894(74)90076-9

[15] Protasio AV, Tsai IJ, Babbage A, Nichol S, Hunt M, Aslett MA et al A systematically improved high quality genome and transcriptome of the human blood fluke Schistosoma mansoni. PLoS Negl Trop Dis. 2012;6(1):e1455 doi: 10.1371/journal.pntd.0001455 22253936 10.1371/journal.pntd.0001455 PMC3254664

[16] Langmead B, Trapnell C, Pop M, Salzberg SL, Ultrafast and memory-efficient alignment of short DNA sequences to the human genome. Genome Biol. 2009 10, R25. 19261174 10.1186/gb-2009-10-3-r25 PMC2690996

[17] Love MI, Huber W, Anders S. Moderated estimation of fold change and dispersion for RNA-seq data with DESeq2. Genome Biol. 2014;15(12):550 ;. doi: 10.1186/s13059-014-0550-8 25516281 10.1186/s13059-014-0550-8 PMC4302049

[18] R Development Core Team (2008). R: A language and environment for statistical computing. R Foundation for Statistical Computing, Vienna, Austria ISBN 3-900051-07-0, http://www.R-project.org.

[19] Sturn A, Quackenbush J, Trajanoski Z. Genesis: Cluster analysis of microarray data. Bioinformatics. 2002 1;18(1):207–8. 11836235 10.1093/bioinformatics/18.1.207

[20] Untergrasser A, Cutcutache I, Koressaar T, Ye J, Faircloth BC, Remm M, Rozen SG Primer3—new capabilities and interfaces. *Nucleic Acids Research*. 2012 40(15):e11521 10.1093/nar/gks596 PMC3424584 22730293

[21] Parker-Manuel SJ, Ivens AC, Dillon GP, Wilson RA. Gene expression patterns in larval Schistosoma mansoni associated with infection of the mammalian host. PLoS Negl Trop Dis. 2011;5(8):e1274 doi: 10.1371/journal.pntd.0001274 21912711 10.1371/journal.pntd.0001274 PMC3166049

[22] Simões MC, Lee J, Djikeng A, Cerqueira GC, Zerlotini A, da Silva-Pereira RA, et al Identification of Schistosoma mansoni microRNAs. BMC Genomics. 2011;12:47 doi: 10.1186/1471-2164-12-47 21247453 10.1186/1471-2164-12-47 PMC3034697

[23] Almeida GT, Amaral MS, Beckedorff FC, Kitajima JP, Demarco R, and Verjovski-Almeida S. Exploring the Schistosoma mansoni adult male transcriptome using RNA-seq.: Exp Parasitol. 2011 9; 132(1):22–31. doi: 10.1016/j.exppara.2011.06.010 21745473 10.1016/j.exppara.2011.06.010

[24] Anderson L, Amaral MS, Beckedorff F, Silva LF, Dazzani B, Oliveira KC et al *Schistosoma mansoni* Egg, Adult Male and Female Comparative Gene Expression Analysis and Identification of Novel Genes by RNA-Seq. PLoS Negl Trop Dis. 2015 12 31;9(12):e0004334 doi: 10.1371/journal.pntd.0004334 26719891 10.1371/journal.pntd.0004334 PMC4699917

[25] Gobert GN, Tran MH, Moertel L, Mulvenna J, Jones MK, McManus DP, Loukas A (2010). Transcriptional Changes in *Schistosoma mansoni* during Early Schistosomula Development and in the Presence of Erythrocytes. *PLoS Neglected Tropical Diseases*, 4(2), e600 http://doi.org/10.1371/journal.pntd.0000600 20161728 10.1371/journal.pntd.0000600 PMC2817720

[26] Sher A, Hall BF, Vadas MA. Acquisition of murine major histocompatibility complex gene products by schistosomula of Schistosoma mansoni. J Exp Med. 1978;148(1):46–57. 97360 10.1084/jem.148.1.46 PMC2184927

[27] Simpson AJ, Payares G, Walker T, Knight M, Smithers SR. The modulation of expression of polypeptide surface antigens on developing schistosomula of Schistosoma mansoni. J Immunol. 1984 11;133(5):2725–30. . 6481169

[28] Ribeiro F, Coelho PMZ, Vieira LQ, Watson DG, Kusel JR. "The effect of praziquantel treatment on glutathione concentration in *Schistosoma mansoni*." Parasitology 1998; vol. 116, no. Pt3, pp. 229–236. 9550216 10.1017/s0031182097002291

[29] Thornhill J, Coelho PM, McVeigh P, Maule A, Jurberg AD, Kusel JR. Schistosoma mansoni cercariae experience influx of macromolecules during skin penetration. Parasitology. 2009;136(11):1257–67. doi: 10.1017/S0031182009990692 19646304 10.1017/S0031182009990692

[30] Thornhill J, Kusel J, Oliviera FA, Ribeiro F, Lima SF, Coelho PM et al Uptake of macromolecules by cercariae during skin penetration and transformation to schistosomula (Schistosoma mansoni). Mem Inst Oswaldo Cruz. 2010;105(4):387–90. 20721480 10.1590/s0074-02762010000400007

[31] Furlong ST, Thibault KS, Morbelli LM, Quinn JJ, Rogers RA. Uptake and compartmentalization of fluorescent lipid analogs in larval Schistosoma mansoni. J Lipid Res. 1995 1;36(1):1–12. 7706934

[32] Daleke DL. Phospholipid flippases. J. Biol. Chem. 2007;282:821–825. doi: 10.1074/jbc.R600035200 17130120 10.1074/jbc.R600035200

[33] Sharom F.J. Flipping and flopping–lipids on the move. IUBMB Life. 2011;63:736–746. doi: 10.1002/iub.515 21793163 10.1002/iub.515

[34] Greenberg RM. Schistosome ABC multidrug transporters: From pharmacology to physiology. *International Journal for Parasitology*: *Drugs and Drug Resistance* 2014;4(3):301–309. doi: 10.1016/j.ijpddr.2014.09.007 25516841 10.1016/j.ijpddr.2014.09.007 PMC4266782

[35] Nyame K, Cummings RD, and Damian RT. Characterization of the high mannose asparagine-linked oligosaccharides synthesized by *Schistosoma mansoni* adult male worms. *Mol*. *Biochem*. *Parasitol*. 1988 28, 265–274. doi: 10.1016/0166-6851(88)90011-4 3386683 10.1016/0166-6851(88)90011-4

[36] Van Die I and Cummings RD. Glycan gimmickry by parasitic helminths: a strategy for modulating the host immune response? *Glycobiology*. 2010 20, 2–12. doi: 10.1093/glycob/cwp140 19748975 10.1093/glycob/cwp140

[37] Jeremias WJ, Melo JRC, Baba EH, Coelho PMZ, Kusel JR (2014) The skin migratory stage of the schistosomulum of Schistosoma mansoni has a surface showing greater permeability and activity in membrane internalization than other forms of skin or mechanical schistosomula. Parasitology. 2015 8;142(9):1143–51. doi: 10.1017/S0031182015000335 Epub 2015 Jun 1. . 26028506 10.1017/S0031182015000335

[38] Ménétret JF, Hegde RS, Heinrich S, Chandramouli P, Ludtke SJ, Rapoport TA et al Architecture of the ribosome-channel complex derived from native membranes. J. Mol. Biol. 2005;348:445–457. doi: 10.1016/j.jmb.2005.02.053 15811380 10.1016/j.jmb.2005.02.053

[39] Schäfer A, Wolf DH. Sec61p is part of the endoplasmic reticulum-associated degradation machinery. *The EMBO Journal* 2009;28(19):2874–2884. doi: 10.1038/emboj.2009.231 19696741 10.1038/emboj.2009.231 PMC2760108

[40] Ye Y, Shibata Y, Yun C, Ron D, Rapoport TA. A membrane protein complex mediates retro-translocation from the ER lumen into the cytosol. Nature. 2004;429:841–7. doi: 10.1038/nature02656 15215856 10.1038/nature02656

[41] Lilley BN, Ploegh HL. A membrane protein required for dislocation of misfolded proteins from the ER. Nature. 2004;429:834–40. doi: 10.1038/nature02592 15215855 10.1038/nature02592

[42] Oda Y, Okada T, Yoshida H, Kaufman RJ, Nagata K, Mori K. Derlin-2 and Derlin-3 are regulated by the mammalian unfolded protein response and are required for ER-associated degradation. J Cell Biol. 2006;172:383–93. doi: 10.1083/jcb.200507057 16449189 10.1083/jcb.200507057 PMC2063648

[43] Goder V, Carvalho P, Rapoport TA. The ER-associated degradation component Der1p and its homolog Dfm1p are contained in complexes with distinct cofactors of the ATPase Cdc48p. *FEBS letters* 2008;582(11):1575–1580. doi: 10.1016/j.febslet.2008.03.056 18407841 10.1016/j.febslet.2008.03.056 PMC2438607

[44] Vembar SS, Brodsky JL. One step at a time: endoplasmic reticulum-associated degradation. *Nature reviews*. *Molecular cell biology* 2008;9(12):944–957. doi: 10.1038/nrm2546 19002207 10.1038/nrm2546 PMC2654601

[45] Kostova Z, Tsai YC, Weissman AM. Ubiquitin ligases, critical mediators of endoplasmic reticulum-associated degradation. Semin. Cell Dev. Biol. 2007;18:770–779. doi: 10.1016/j.semcdb.2007.09.002 17950636 10.1016/j.semcdb.2007.09.002 PMC2200800

[46] McNair A, Zemzoumi K, Lütcke H, Guillerme C, Boitelle A, Capron A et al Cloning of a signal-recognition-particle subunit of Schistosoma mansoni. Parasitol Res. 1995;81(2):175–7. . 7731929 10.1007/BF00931628

[47] Zimmermann R, Eyrisch S, Ahmad M, Helms V. Protein translocation across the ER membrane. Biochim Biophys Acta. 2011 3;1808(3):912–24. doi: 10.1016/j.bbamem.2010.06.015 Epub 2010 Jun 27. Review. . 20599535 10.1016/j.bbamem.2010.06.015

[48] Palty R, Raveh A, Kaminsky I, Meller R, Reuveny E. SARAF inactivates the storeoperated calcium entry machinery to prevent excess calcium refilling. Cell. 2012 4 13;149(2):425–38. doi: 10.1016/j.cell.2012.01.055 Epub 2012 Mar 29. . 22464749 10.1016/j.cell.2012.01.055

[49] Shoda M, Misumi Y, Yamamoto A, Yano A, Nakamura N, Ikehara Y. Identification and characterization of a novel Golgi protein, GCP60, that interacts with the integral membrane protein giantin. J Biol Chem. 2001 11 30;276(48):45298–306. doi: 10.1074/jbc.M108961200 11590181 10.1074/jbc.M108961200

[50] Matsuda A, Suzuki Y, Honda G, Muramatsu S, Matsuzaki O, Nagano Y et al "Large-scale identification and characterization of human genes that activate NF-kappaB and MAPK signaling pathways". *Oncogene*. 2003 5 22 (21): 3307–3318. doi: 10.1038/sj.onc.1206406 12761501 10.1038/sj.onc.1206406

[51] Sbodio JI, Hicks SW, Simon D, Machamer CE. GCP60 preferentially interacts witha caspase-generated golgin-160 fragment. J Biol Chem. 2006 9 22;281(38):27924–31. Epub 2006 Jul 26. doi: 10.1074/jbc.M603276200 16870622 10.1074/jbc.M603276200

[52] Fitzpatrick JM, Johnston DA, Williams GW, Williams DJ, Freeman TC, Dunne DW et al An oligonucleotide microarray for transcriptome analysis of *Schistosoma mansoni* and its application/use to investigate gender-associated gene expression. Mol Biochem Parasitol 2005;141:1–13. []. doi: 10.1016/j.molbiopara.2005.01.007 15811522 10.1016/j.molbiopara.2005.01.007

[53] Moertel L, McManus DP, Piva TJ, Young L, McInnes RL, Gobert GN. Oligonucleotide microarray analysis of strain- and gender-associated gene expression in the human blood fluke, *Schistosoma japonicum*. Mol Cell Probes 2006;20:280–9. doi: 10.1016/j.mcp.2006.02.002 16647836 10.1016/j.mcp.2006.02.002

[54] Aragon AD, Imani RA, Blackburn VR, Cunningham C. Microarray based analysis of temperature and oxidative stress induced messenger RNA in *Schistosoma mansoni*. *Molecular and biochemical parasitology* 2008;162(2):134–141. doi: 10.1016/j.molbiopara.2008.08.004 18775750 10.1016/j.molbiopara.2008.08.004 PMC2591067

[55] Chen JH, Zhang T, Ju C, Xu B, Lu Y, Mo XJ et al An integrated immunoproteomics and bioinformatics approach for the analysis of Schistosoma japonicum tegument proteins. J Proteomics. 2014 2 26;98:289–99. doi: 10.1016/j.jprot.2014.01.010 Epub 2014 Jan 19. 24448400 10.1016/j.jprot.2014.01.010

[56] Fischer G, Bang H. The refolding of urea-denatured ribonuclease A is catalyzedby peptidyl-prolyl cis-trans isomerase. Biochim Biophys Acta. 1985 3 22;828(1):39–42. . 3882150 10.1016/0167-4838(85)90006-8

[57] Lang K, Schmid FX, Fischer G. Catalysis of protein folding by prolylisomerase. Nature. 1987 9 17–23;329(6136):268–70. doi: 10.1038/329268a0 3306408 10.1038/329268a0

[58] Fischer G, Wittmann-Liebold B, Lang K, Kiefhaber T, Schmid FX. Cyclophilin andpeptidyl-prolyl cis-trans isomerase are probably identical proteins. Nature. 1989 2 2;337(6206):476–8. doi: 10.1038/337476a0 2492638 10.1038/337476a0

[59] Bout D, Deslèe D, Capron A. Antischistosomal effect of cyclosporin A: cure and prevention of mouse and rat schistosomiasis mansoni. *Infection and Immunity*. 1986;52(3):823–827. 3086233 10.1128/iai.52.3.823-827.1986 PMC260933

[60] Bout DT, Deslèe D, Capron AR. Protection against schistosomiasis produced bycyclosporin A. Am J Trop Med Hyg. 1984 1;33(1):185–6. . 6696178 10.4269/ajtmh.1984.33.185

[61] Brannan LR, Chappell LH, Woo J, Thomson AW. Anti-schistosomal activity of cyclosporin A: studies on murine spleen cells and the influence of a cyclosporin antagonist on resistance to infection. *Immunology*. 1989;67(3):382–387. 2503438 PMC1385357

[62] Munro GH, Brannan LR, Chappell LH, Thomson AW, McLaren DJ. The larvicidalactivity of cyclosporin A against *Schistosoma mansoni* in mice. Parasitology. 1991 2;102 Pt 1:57–63. . 1903880 10.1017/s0031182000060340

[63] Koletsky AJ, Harding MW, Handschumacher RE. Cyclophilin: distribution andvariant properties in normal and neoplastic tissues. J Immunol. 1986 8 1;137(3):1054–9. . 3522735

[64] Horn M, Fajtová P, Rojo Arreola L, Ulrychová L, Bartošová-Sojková P, Franta Z, et al Trypsin- and Chymotrypsin-like serine proteases in schistosoma mansoni- 'the undiscovered country'. PLoS Negl Trop Dis. 2014 3 27;8(3):e2766 doi: 10.1371/journal.pntd.0002766 ;. 24676141 10.1371/journal.pntd.0002766 PMC3967958

[65] Rawlings ND, Barrett AJ, Bateman A, MEROPS: the database of proteolytic enzymes, their substrates and inhibitors. Nucleic Acids Res 2012 40: D343–D350. 22086950 10.1093/nar/gkr987 PMC3245014

[66] Hanks SK, Quinn AM, and Hunter T, “The protein kinase family: conserved features and deduced phylogeny of the catalytic domains,” Science, 1988 241(4861), 42–52. 3291115 10.1126/science.3291115

[67] Manning G, Whyte DB, Martinez R, Hunter T, Sudarsanam S. The protein kinase complement of the human genome. Science. 2002 12 6;298(5600):1912–34. Review. doi: 10.1126/science.1075762 12471243 10.1126/science.1075762

[68] Loeffler DA, Lundy SK, Singh KP, Gerard HC, Hudson AP, and Boros DL—Soluble Egg Antigens from *Schistosoma mansoni* Induce Angiogenesis-Related Processes by Up-Regulating Vascular Endothelial Growth Factor in Human Endothelial Cells. J Infect Dis. 2002 185 (11): 1650–1656 doi: 10.1086/340416 12023772 10.1086/340416

[69] Yamamoto C, Yagi S, Hori T, Iida T, Taniguchi K, Isaji S et al Significance of portal venous VEGF during liver regeneration after hepatectomy. J Surg Res. 2010 4;159(2):e37–43. doi: 10.1016/j.jss.2008.11.007 19394640 10.1016/j.jss.2008.11.007

[70] Harvey RP. NK-2 homeobox genes and heart development. Dev Biol. 1996 9 15;178(2):203–16. Review. doi: 10.1006/dbio.1996.0212 8812123 10.1006/dbio.1996.0212

[71] Price M, Lazzaro D, Pohl T, Mattei MG, Rüther U, Olivo JC et al Regional expression of the homeobox gene Nkx-2.2 in the developing mammalian forebrain. Neuron. 1992 2;8(2):241–55. . 1346742 10.1016/0896-6273(92)90291-k

[72] Rudnick A, Ling TY, Odagiri H, Rutter WJ, German MS. Pancreatic beta cells express a diverse set of homeobox genes. *Proceedings of the National Academy of Sciences of the United States of America*. 1994;91(25):12203–12207. 7991607 10.1073/pnas.91.25.12203 PMC45405

[73] Ceulemans H, Stalmans W, Bollen M. Regulator-driven functional diversification of protein phosphatase-1 in eukaryotic evolution. Bioessays. 2002 4;24(4):371–81. Review. doi: 10.1002/bies.10069 11948623 10.1002/bies.10069

[74] Daher W, Cailliau K, Takeda K, Pierrot C, Khayath N, Dissous C et al Characterization of Schistosoma mansoni Sds homologue, a leucine-rich repeat protein that interacts with protein phosphatase type 1 and interrupts a G2/M cell-cycle checkpoint. Biochem J. 2006 4, 15;395(2):433–41. ; doi: 10.1042/BJ20051597 16411888 10.1042/BJ20051597 PMC1422774

[75] Beullens M, Bollen M. The protein phosphatase-1 regulator NIPP1 is also a splicing factor involved in a late step of spliceosome assembly. J Biol Chem.2002 5 31;277(22):19855–60. Epub 2002 Mar 21. Bioinformatics. 2002 Jan;18(1):207–8. doi: 10.1074/jbc.M200847200 11909864 10.1074/jbc.M200847200

[76] Parker L, Gross S, Beullens M, Bollen M, Bennett D, Alphey L. Functional interaction between nuclear inhibitor of protein phosphatase type 1 (NIPP1) and protein phosphatase type 1 (PP1) in Drosophila: consequences of over-expression of NIPP1 in flies and suppression by co-expression of PP1. *Biochemical Journal*. 2002;368(Pt 3):789–797. doi: 10.1042/BJ20020582 12358598 10.1042/BJ20020582 PMC1223049

[77] Lee EF, Young ND, Lim NT, Gasser RB, Fairlie WD. Apoptosis in schistosomes: toward novel targets for the treatment of schistosomiasis. Trends Parasitol. 2014 2;30(2):75–84. doi: 10.1016/j.pt.2013.12.005 Epub 2014 Jan 3. Review. . 24393571 10.1016/j.pt.2013.12.005

[78] Mosavi LK, Cammett TJ, Desrosiers DC, Peng Z. The ankyrin repeat as molecular architecture for protein recognition. *Protein Science *: *A Publication of the Protein Society*. 2004;13(6):1435–1448. doi: 10.1110/ps.03554604 15152081 10.1110/ps.03554604 PMC2279977

[79] Draper BW, Mello CC, Bowerman B, Hardin J and Priess JR. MEX-3 is a KH domain protein that regulates blastomere identity in early C. elegans embryos. Cell, 1996 87, 205–216. 8861905 10.1016/s0092-8674(00)81339-2

[80] Ciosk R, DePalma M and Priess JR (2006) Translational regulators maintain totipotency in the Caenorhabditis elegans germline. Science, 311, 851–853. doi: 10.1126/science.1122491 16469927 10.1126/science.1122491

[81] Buchet-Poyau K, Courchet J, Le Hir H, Séraphin B, Scoazec JY, Duret L et al Identification and characterization of human Mex-3 proteins, a novel family of evolutionarily conserved RNA-binding proteins differentially localized to processing bodies. Nucleic Acids Res. 2007;35(4):1289–300. Epub 2007 Jan 31. ; doi: 10.1093/nar/gkm016 17267406 10.1093/nar/gkm016 PMC1851655

[82] Takada H, Kawana T, Ito Y, Kikuno RF, Mamada H, Araki T et al The RNA-binding protein Mex3b has a fine-tuning system for mRNA regulation in early Xenopus development. Development. 2009 7;136(14):2413–22. doi: 10.1242/dev.029165 . 19542354 10.1242/dev.029165

[83] Abreu FC, Pereira RV, Oliveira VF, Gomes Mde S, Jannotti-Passos LK, Borges WC et al Characterization of export receptor exportins (XPOs) in the parasiteSchistosoma mansoni. Parasitol Res. 2013 12;112(12):4151–9. doi: 10.1007/s00436-013-3606-x Epub 2013 Sep 8. . 24013345 10.1007/s00436-013-3606-x

[84] Dovey HF, McKerrow JH, Aldritt SM, Wang CC. Purification and characterization of hypoxanthine-guanine phosphoribosyltransferase from Schistosoma mansoni. Apotential target for chemotherapy. J Biol Chem. 1986 1 15;261(2):944–8. . 3941107

[85] Craig SP, McKerrow JH, Newport GR, Wang CC. Analysis of cDNA encoding the hypoxanthine-guanine phosphoribosyltransferase (HGPRTase) of Schistosoma mansoni; a putative target for chemotherapy. *Nucleic Acids Research*. 1988;16(14B):7087–7101. 3136439 10.1093/nar/16.14.7087 PMC338353

[86] Murray AW, Elliott DC, Atkinson MR. Nucleotide biosynthesis from preformedpurines in mammalian cells: regulatory mechanisms and biological significance. Prog Nucleic Acid Res Mol Biol. 1970;10:87–119. Review. . 4910307 10.1016/s0079-6603(08)60562-0

