# Supplementary material for: Comparative sequence analysis reveals regulation of genes in developing schistosomula of Schistosoma mansoni exposed to host portal serum
Source: PLoS One. 2017 Jun 16;12(6):e0178829. doi: 10.1371/journal.pone.0178829 (PMC5473564; doi:10.1371/journal.pone.0178829)
Supplement: S1 Table — (DOCX) [file pone.0178829.s001.docx]

| Table S1: List of oligos used in qPCR of samples SPO3H and SPO12H | | |
| --- | --- | --- |
| **Description** | **Oligo ID** | **Sequence** |
| Smp_157070.1 cysteine rich with egf domains protein | F1570 | aaacaaacctggttcttacg |
|  | R1570 | tcctctttggtatccacttg |
|  | O1570 | ccgactgcaaacgattgtga |
| Smp_027990.1 homeobox protein nk 2 ; neural gene activation | F0279 | cttcttcatggtacggtgtt |
|  | R0279 | tttgttacacccattaaacg |
|  | O0279 | agccgctgttgctaatgacc |
| Smp_177040.1 gpi mannosyltransferase 2 | F1770 | tcgctccctttcttattg |
|  | R1770 | aaggcgaactggtattga |
|  | O1770 | cgcgatcgtttcacaccata |
| Smp_019060.1 sec61 beta subunit | F0190 | atcctctgcgtcatcaaa |
|  | R0190 | cgactggtcgtctagctg |
|  | O0190 | tgctactgcctcaccaaggg |
| Smp_089000.1 translocon associated protein subunit delta; translocon associated protein delta subunit | F0890 | ttaggccggttacctatt |
|  | R0890 | acgagcaactggttgaag |
|  | O0890 | caagcgaagcagttctttcca |
| Smp_042790.1 dolichol phosphate mannosyltransferase | F0427 | gcccacctacaatgagaa |
|  | R0427 | tgccatctggactgttgt |
|  | O0427 | aaagtggctttccctacgaact |
| Smp_172110.1 protein disulfide isomerase A6 | F1721 | tggaaactattcgccact |
|  | R1721 | tggaaagcccttaacaga |
|  | O1721 | tgctgcggttgattcagaaa |
| Smp_083080.1 Activator of 90 kDa heat shock protein ATPase | F0830 | atactgccgatggaatca |
|  | R0830 | ggcctcagatcgtgtaaa |
|  | O0830 | ttcttctgtactccggatgaccttt |
| Smp_134570.1 hypothetical protein | F1345 | ggtgcaacaaacagtcca |
|  | R1345 | ttgtccattccattgagg |
|  | O1345 | tgatcaattcaacaatttcaccattc |
| Smp_130260.1 hypothetical protein | F1302 | tctacaagtccacgatcaaa |
|  | R1302 | ctagagccggaattcaaa |
|  | O1302 | aagcctcaatatcaacagcacca |
| Smp_103560.1 hypoxanthine guanine phosphoribosyltransferase | F1035 | gcgacatcgatcacactt |
|  | R1035 | caacggacattggtagga |
|  | O1035 | atgggcttgaacgcactgtc |
| Smp_024390.1 microsomal signal peptidase 25 kDa subunit | F0243 | acggtgtgtgacggtatt |
|  | R0243 | aacccgtcgtattgactt |
|  | O0243 | agtcaggacgctttccgttg |
| Smp_079230.1 immunophilin FK506 binding protein FKBP12, putative | F0792 | acgaattccaaagaaagg |
|  | R0792 | acaccttcatcccaacct |
|  | O0792 | tcccatccatgagggtacca |
| Smp_079420.1 ankyrin repeat domain containing protein 42 | F0794 | cgatgccttgaatgaact |
|  | R0794 | gtagcaacttcgcgtctt |
|  | O0794 | cgttcacattccaatgttgcttt |
| Smp_145540.1 muscarinic acetylcholine receptor | F1455 | tcacgttatgggaccttt |
|  | R1455 | ctcatcaggtgcttgctt |
|  | O1455 | caatccattagagcaatcttcgg |
| Smp_175790.1 phospholipid translocating ATPase | F1757 | atgtgaacaaccaaatgaaa |
|  | R1757 | aacatgtttcgtcaccatc |
|  | O1757 | tgctcctgttgctcctgatga |
|  |  |  |
|  |  |  |
| Smp_136310.1 sodium bile acid cotransporter | F1363 | catctacggtcggttctt |
|  | R1363 | ccaatttcggtttccata |
|  | O1363 | cctgcattaatgggcttaggtc |
| Smp_053420.1 fermitin family 1 | F0534 | ccacgtagcaacaataagaa |
|  | R0534 | caccggaatcttcatcag |
|  | O0534 | ccttctgtcaatgaagatttcgc |
| Smp_007270.1 smoothelin | F0072 | tgactggaaatgcgtctt |
|  | R0072 | gagcagctttaggaagga |
|  | O0072 | attaccggcgtttccatttg |
| Smp_900000 – cicloxigenase 1 COX1 | FCO1 | tacggttggtggtgtcacag |
|  | RCO1 | acggccatcaccatactagc |
|  | OCO1 | tttcatgatacttggtttgttgttgc |
