# Supplementary material for: Comparative sequence analysis reveals regulation of genes in developing schistosomula of Schistosoma mansoni exposed to host portal serum
Source: PLoS One. 2017 Jun 16;12(6):e0178829. doi: 10.1371/journal.pone.0178829 (PMC5473564; doi:10.1371/journal.pone.0178829)
Supplement: S2 Table — (DOCX) [file pone.0178829.s002.docx]

| Table S2: Genes differentially expressed in schistosomula cultured of 12hours in portal serum | | | |
| --- | --- | --- | --- |
|  |  |  |  |
| Gene identifier | Description | Adj. P-value | Fold Change |
| Smp_002150_1 | trypsin-like serine protease Mastin | 7.99E-04 | 2.92 |
| Smp_070020_1 | rhoptry associated membrane antigen | 1.47E-03 | 2.91 |
| Smp_042400_1 | arginine rich, mutated in early stage tumors | 1.61E-03 | 2.86 |
| Smp_030300_3 | endoplasmin | 3.17E-10 | 2.8 |
| Smp_157070_1 | cysteine rich with egf domains protein | 1.67E-03 | 2.76 |
| Smp_134570_1 | hypothetical protein | 2.10E-05 | 2.7 |
| Smp_030300_4 | endoplasmin | 2.01E-08 | 2.64 |
| Smp_024390_3 | microsomal signal peptidase 25 kDa subunit | 4.02E-03 | 2.64 |
| Smp_088950_1 | Hypoxia up regulated protein | 5.87E-04 | 2.64 |
| Smp_049550_1 | 78 kDa glucose regulated protein | 1.57E-06 | 2.61 |
| Smp_130260_1 | hypothetical protein | 6.41E-03 | 2.6 |
| Smp_024390_1 | microsomal signal peptidase 25 kDa subunit | 3.99E-03 | 2.58 |
| Smp_172110_1 | protein disulfide isomerase A6 | 2.09E-06 | 2.4 |
| Smp_095400_1 | hypothetical protein | 1.54E-02 | 2.36 |
| Smp_024390_2 | microsomal signal peptidase 25 kDa subunit | 1.75E-02 | 2.33 |
| Smp_059480_1 | tryparedoxin peroxidase | 4.47E-03 | 2.32 |
| Smp_202980_1 | hypothetical protein | 2.85E-02 | 2.29 |
| Smp_030370_1 | calreticulin | 5.01E-09 | 2.24 |
| Smp_042140_1 | tegument-allergen-like protein | 4.34E-03 | 2.23 |
| Smp_148790_1 | laminin subunit beta 1 | 3.77E-02 | 2.21 |
| Smp_007450_1 | heat shock protein 67b2 | 6.41E-03 | 2.18 |
| Smp_079770_2 | protein disulfide-isomerase ER-60 precursor | 2.85E-04 | 2.17 |
| Smp_177040_1 | gpi mannosyltransferase 2 | 9.39E-04 | 2.16 |
| Smp_204760_1 | Asparagine rich protein | 4.85E-02 | 2.14 |
| Smp_083080_2 | Activator of 90 kDa heat shock protein ATPase | 1.10E-04 | 2.12 |
| Smp_063330_1 | hypothetical protein | 2.92E-02 | 2.12 |
| Smp_027990_1 | homeobox protein nk 2 | 5.74E-03 | 2.11 |
| Smp_079770_1 | protein disulfide-isomerase ER-60 precursor | 7.63E-04 | 2.09 |
| Smp_089000_1 | translocon associated protein subunit delta | 2.73E-02 | 2.05 |
| Smp_155510_1 | transmembrane protein 66 | 2.87E-03 | 2.05 |
| Smp_103560_1 | hypoxanthine guanine phosphoribosyltransferase | 4.27E-02 | 2.05 |
| Smp_079230_1 | immunophilin FK506 binding protein FKBP12 | 4.68E-02 | 2.03 |
| Smp_079420_1 | ankyrin repeat domain containing protein 42 | 5E-02 | 2.02 |
| Smp_042790_1 | dolichol phosphate mannosyltransferase | 5E-02 | 2.02 |
| Smp_019060_1 | sec61 beta subunit | 1.67E-03 | 2.01 |
| Smp_097380_1 | heat shock 10 kDa protein 1 | 2.03E-03 | 2 |
| Smp_004780_3 | immunophilin | 2.85E-02 | 1.99 |
| Smp_020800_1 | cysteine and histidine rich domain containing protein | 2.01E-02 | 1.99 |
| Smp_024370_1 | hypothetical protein | 2.48E-02 | 1.97 |
| Smp_004780_2 | immunophilin | 5.74E-03 | 1.96 |
| Smp_011270_1 | sj ts4 protein | 3.27E-02 | 1.95 |
| Smp_157360_1 | E3 ubiquitin protein ligase synoviolin B | 3.27E-02 | 1.9 |
| Smp_079310_1 | transmembrane emp24 domain containing protein | 2.16E-02 | 1.89 |
| Smp_176200_2 | superoxide dismutase [Cu-Zn] | 2.92E-02 | 1.88 |
| Smp_083080_1 | Activator of 90 kDa heat shock protein ATPase | 3.58E-03 | 1.88 |
| Smp_004780_1 | immunophilin | 2.46E-02 | 1.87 |
| Smp_011700_1 | proto oncogene tyrosine protein kinase ROS | 4.86E-04 | 1.84 |
| Smp_059350_1 | der1 protein derlin | 5E-02 | 1.84 |
| Smp_157940_1 | Nuclear inhibitor of protein phosphatase 1 | 4.55E-02 | 1.83 |
| Smp_011690_1 | proto oncogene tyrosine protein kinase ROS | 3.82E-02 | 1.83 |
| Smp_032760_1 | ribosomal protein S11 | 2.01E-02 | 1.74 |
| Smp_119920_1 | ribosomal protein S16 | 7.54E-03 | 1.74 |
| Smp_210370_1 | ormdl protein | 2.23E-02 | 1.72 |
| Smp_031570_1 | ribosomal protein L18 | 2.46E-02 | 1.71 |
| Smp_167230_1 | golgi resident protein gcp60 | 2.85E-02 | 1.7 |
| Smp_072950_1 | Transmembrane emp24 domain containing protein 4 | 5.19E-03 | 1.69 |
| Smp_064860_1 | stress induced phosphoprotein 1 | 3.30E-02 | 1.69 |
| Smp_092810_1 | transmembrane protein 33 | 3.24E-02 | 1.68 |
| Smp_194090_1 | subfamily S1A unassigned peptidase | 3.30E-02 | 1.65 |
| Smp_124820_1 | chromosome region maintenance protein | 2.12E-02 | 1.65 |
| Smp_171190_1 | MEG-8 family | 1.78E-02 | 1.64 |
| Smp_090120_2 | alpha tubulin | 3.28E-02 | 1.61 |
| Smp_207010_1 | signal recognition particle 72 kDa subunit | 1.31E-02 | 1.57 |
| Smp_009310_1 | plasminogen activator inhibitor 1 RNA binding | 1.32E-02 | 1.51 |
| Smp_040790_1 | peptidyl prolyl cis trans isomerase B | 2.97E-02 | 1.49 |
| Smp_210780_1 | Loss of heterozygosity 11 chromosomal region 2 | 4.26E-02 | 0.73 |
| Smp_078160_1 | RB1 inducible coiled coil protein 1 | 4.68E-02 | 0.69 |
| Smp_123320_1 | chromodomain helicase DNA binding protein | 9.25E-03 | 0.68 |
| Smp_176060_1 | e3 ubiquitin protein ligase ubr2 | 4.98E-03 | 0.63 |
| Smp_179660_1 | hypothetical protein | 2.85E-02 | 0.62 |
| Smp_171720_1 | lipid phosphate phosphohydrolase 1 | 4.27E-02 | 0.62 |
| Smp_123260_1 | AN1 type zinc finger and ubiquitin | 7.99E-04 | 0.62 |
| Smp_012720_1 | hypothetical protein | 3.58E-03 | 0.61 |
| Smp_024900_1 | retinoblastoma protein 1 | 2.46E-03 | 0.61 |
| Smp_014400_6 | enhancer of polycomb | 2.86E-02 | 0.6 |
| Smp_043030_1 | hexokinase | 2.48E-02 | 0.6 |
| Smp_145080_1 | hypothetical protein | 2.86E-03 | 0.6 |
| Smp_032280_1 | RNA binding protein MEX3B | 1.78E-03 | 0.6 |
| Smp_081450_1 | ectonucleoside triphosphate diphosphohydrolase | 2.39E-02 | 0.59 |
| Smp_126510_1 | hypothetical protein | 4.06E-02 | 0.57 |
| Smp_094890_1 | hypothetical protein | 2.58E-03 | 0.57 |
| Smp_169040_1 | lysophospholipid acyltransferase 5 | 1.42E-02 | 0.56 |
| Smp_153290_1 | cement protein 3B variant 3 | 2.85E-02 | 0.56 |
| Smp_090890_1 | proto oncogene serine:threonine protein kinase | 8.74E-05 | 0.56 |
| Smp_071380_2 | zinc finger protein | 3.21E-02 | 0.56 |
| Smp_073340_1 | cyclin dependent kinase 5 | 2.16E-02 | 0.56 |
| Smp_150030_1 | hypothetical protein | 3.59E-02 | 0.55 |
| Smp_133830_1 | hypothetical protein | 7.16E-04 | 0.53 |
| Smp_159090_1 | hypothetical protein | 2.69E-02 | 0.52 |
| Smp_052230_1 | Papilin | 4.02E-03 | 0.52 |
| Smp_180810_1 | Papilin | 4.79E-07 | 0.51 |
| Smp_180690_1 | tiptop protein | 3.45E-02 | 0.51 |
| Smp_135710_1 | forkhead box protein K1 | 1.31E-02 | 0.51 |
| Smp_136310_1 | sodium bile acid cotransporter | 2.85E-02 | 0.49 |
| Smp_053420_1 | fermitin family 1 | 7.16E-04 | 0.49 |
| Smp_007270_1 | smoothelin | 4.34E-03 | 0.49 |
| Smp_037610_1 | hypothetical protein | 3.08E-02 | 0.49 |
| Smp_121940_1 | FERM domain containing protein 5 | 6.41E-03 | 0.49 |
| Smp_159620_1 | BTB:POZ domain containing protein KCTD20 | 5.20E-11 | 0.47 |
| Smp_176110_1 | hypothetical protein | 4.40E-03 | 0.47 |
| Smp_175790_1 | phospholipid translocating ATPase | 7.63E-04 | 0.42 |
| Smp_004590_1 | hypothetical protein | 5.12E-05 | 0.37 |
| Smp_145540_1 | muscarinic acetylcholine receptor | 7.16E-04 | 0.34 |
| Differentially expressed genes with adjusted p-value <0.05. | | | |
|  |  |  |  |
